# Leptin and Its Relation to Obesity and Insulin in the SHR/N-corpulent Rat, A Model of Type II Diabetes Mellitus

**DOI:** 10.1155/EDR.2001.217

**Published:** 2001

**Authors:** Manuel T. Velasquez, Sam J. Bhathena, Carl T. Hansen

**Affiliations:** 1 Division of Renal Diseases and Hypertension Department of Medicine George Washington University Medical Center 2150 Pennsylvania Avenue NW Washington, DC 20037 USA; 2 Beltsville Human Nutrition Research Center Agricultural Research Service US Department of Agriculture Beltsville Maryland USA; 3 National Institutes of Health Animal Genetic Resource Bethesda Maryland USA

## Abstract

The spontaneously hypertensive/NIH-corpulent (SHR/N-cp) rat is a genetic animal model that exhibits obesity, metabolic features of hyperinsulinemia, hyperglycemia, and hyperlipidemia, which are characteristic of type II diabetes and mild hypertension. To determine the role of leptin, the protein product of the *ob* gene, in the development of obesity and diabetes in this model, we measured steady-state circulating levels of leptin in obese and lean SHR/N-cp rats and examined the relation between plasma leptin levels and metabolic variables at the stage of established obesity in these animals. Mean fasting plasma leptin concentration was 8-fold higher in obese than in lean rats (p<0.01). This was associated with a 6-fold elevation in plasma insulin in the obese group. Fasting levels of plasma glucose, cholesterol, and triglyceride were all significantly higher in obese rats than in lean controls. Spearman correlation analysis showed a significant positive correlation between plasma leptin concentration and body weight among the animals (r=0.73, p<0.01). Similarly, plasma insulin concentration was significantly correlated with BW in all animals (r=0.54, p<0.05). There was also a significant positive.correlation between plasma leptin and plasma insulin in the entire group (r=0.70, p<0.01). However, this relationship was significant only for lean rats but not for obese rats (r=0.59, p<0.05 for lean rats, and r=0.23, p=NS, for obese rats). Plasma leptin also correlated positively with fasting plasma glucose (r=0.75, p<0.05), total cholesterol (r=0.63, p<0.05), and triglyceride (r=0.67, p <0.05). The marked elevation of plasma leptin in obese SHR/N-cp rats suggests that obesity in this animal model is related to up-regulation of the ob gene. Circulating leptin appears to be one of the best biological markers of obesity and that hyperleptinemia is closely associated with several metabolic risk factors related to insulin resistance in the diabesity syndrome.


*Int. Jnl. Experimental Diab. Res.*, Vol. 2, pp. 217–223
Reprints available directly from the publisher
Photocopying permitted by license only

© 2001 OPA (Overseas Publishers Association) N.V.
Published by license under
the Harwood Academic Publishers imprint,
part of Gordon and Breach Publishing,
a member of the Taylor & Francis Group.
Printed in the U.S.A.

### Leptin and Its Relation to Obesity and Insulin in the SHR/N-corpulent Rat, A Model of Type II Diabetes Mellitus

MANUEL T. VELASQUEZ[a,*], SAM J. BHATHENA[b] and CARL T. HANSEN[c]

[a]*Division of Renal Diseases and Hypertension, Department of Medicine, George Washington University Medical Center, 2150 Pennsylvania Avenue, NW, Washington, DC 20037;* [b]*Beltsville Human Nutrition Research Center, Agricultural Research Service, US Department of Agriculture, Beltsville, Maryland;*
[c]*National Institutes of Health, Animal Genetic Resource, Bethesda, Maryland*

*(Received 1 November 2000; In final form 1 August 2001)*

**The spontaneously hypertensive/NIH-corpulent (SHR/N-cp) rat is a genetic animal model that exhibits obesity, metabolic features of hyperinsulinemia, hyperglycemia, and hyperlipidemia, which are characteristic of type II diabetes and mild hypertension. To determine the role of leptin, the protein product of the *ob* gene, in the development of obesity and diabetes in this model, we measured steady-state circulating levels of leptin in obese and lean SHR/N-cp rats and examined the relation between plasma leptin levels and metabolic variables at the stage of established obesity in these animals. Mean fasting plasma leptin concentration was 8-fold higher in obese than in lean rats ($p<0.01$). This was associated with a 6-fold elevation in plasma insulin in the obese group. Fasting levels of plasma glucose, cholesterol, and triglyceride were all significantly higher in obese rats than in lean controls. Spearman correlation analysis showed a significant positive correlation between plasma leptin concentration and body weight among the animals ($r=0.73$, $p<0.01$). Similarly, plasma insulin concentration was significantly correlated with BW in all animals ($r=0.54$, $p<0.05$). There was also a significant positive correlation between plasma leptin and plasma insulin in the entire group ($r=0.70$, $p<0.01$). However, this relationship was significant only for lean rats but not for obese rats ($r=0.59$, $p<0.05$ for lean rats, and $r=0.23$, p = NS, for obese rats). Plasma leptin also correlated positively with fasting plasma glucose ($r=0.75$, $p<0.05$), total cholesterol ($r=0.63$, $p<0.05$), and triglyceride ($r=0.67$, $p<0.05$). The marked elevation of plasma leptin in obese SHR/N-cp rats suggests that obesity in this animal model is related to up-regulation of the ob gene. Circulating leptin appears to be one of the best biological markers of obesity and that hyperleptinemia is closely associated with several metabolic risk factors related to insulin resistance in the diabesity syndrome.**

*Keywords*: Insulin; Leptin; Lipids; Type II diabetes mellitus; Obesity; SHR/N-cp rats

### INTRODUCTION

Obesity and type II diabetes or non-insulin-dependent diabetes mellitus (NIDDM) are two prevalent disorders that have become a major public health concern in industrialized countries because of their frequent association with cardiovascular risk factors, namely, hypertension, dyslipidemia, atherosclerosis, and coronary heart disease.[1–3] These two conditions often coexist and have in common the metabolic features of hyperinsulinemia and glucose intolerance.[4, 5] In fact, both are considered as disorders of insulin resistance.[6] Since the identification of the obese (*ob*) gene in 1994,[7] it became apparent that obesity in

*Corresponding author.

humans as well as in animals is associated with abnormalities in the expression this gene and its protein product, leptin. In the *ob/ob* mouse, a mutation in the *ob* gene causes a deficiency of leptin and leads to increased food intake, reduced energy expenditure, and marked obesity.[8–10] Administration of leptin to these mice reduces food intake, body weight, and adiposity.[7–10] In contrast to findings in *ob/ob* mice, *ob* gene expression and circulating leptin levels are increased in obese humans and both factors correlate positively with the amount of body fat.[11–13] Similarly, leptin mRNA expression and circulating leptin have also been found to be elevated in most other rodent models of obesity, including diabetic (*db/db*) mice, Wistar fatty rats, Zucker (*fa/fa* rats), and ventromedial hypothalamus-lesioned mice.[14–17]

The spontaneously hypertensive/NIH-corpulent (SHR/N-cp) rat is a genetic animal model that exhibits obesity, metabolic features of hyperinsulinmia, hyperglycemia, and hyperlipidemia characteristic of NIDDM, and mild hypertension.[18–20] Unlike other rodent models of obesity, the SHR/N-cp rat is a congenic strain. This was achieved by an initial mating of a male obese spontaneously hypertensive (Koletsky) rat which was heterozygous for the *cp* gene with a spontaneously hypertensive rat (SHR) of the Okamoto strain, followed by multiple rounds of back-crossing of the progeny to the SHR strain.[19,20] Since the *cp* gene is autosomal recessive and the corpulent homozygotes (*cp/cp*) do not reproduce, heterozygotes (*cp*/+) were used in backcrossing to produce the next generation of animals. A minimum of ten backcrosses was carried out to eliminate the non-cp genes of the Koletsky strain. Once the congenic strains were established, mating of heterozygotes yields three genotypes but only two phenotypes: homozygous (*cp/cp*) corpulent, heterozygous (cp/+) lean, and homozygous (+/+) lean, in a ratio of 1:2:1. Corpulent homozygotes, unlike their lean littermates, are characterized by central (abdominal) obesity, hyperglycemia, hyperinsulinemia, hyperlipidemia, and mild hypertension.

The role of leptin secretion and its relationship to hyperinsulinemia in this strain has not been examined. This is of interest since leptin has been suggested to play a causative role in insulin resistance associated with obesity.[21] Consequently, we have studied the SHR/N-*cp* rat to examine the effect of genetic obesity on leptin in this model. Specifically, we determined steady-state circulating levels of leptin in corpulent and lean SHR/N-cp rats and examined the relation between plasma leptin levels and metabolic parameters at the stage of established obesity in these animals.

### MATERIALS AND METHODS

Male SHR/N-*cp* rats were obtained from the National Institutes of Health at 5–6 weeks of age. At this age, obesity is already evident in SHR/N-corpulent (*cp/cp*) rats as suggested by higher body weight than their lean littermates and increased fat in the abdomen. All procedures for the study were approved by the Institutional Animal Care and Use Committees of the George Washington University, Washington, D.C., and Agricultural Research Service, U.S. Department of Agriculture, Beltsville, Maryland.

All animals were housed individually in stainless steel wire cages with controlled temperature (21 to 25°C) and relative humidity (40 to 50%) and maintained on a reverse 12-hour dark (0900 to 2100 hr) and light (2100 to 0900 hr) cycle. Rats were fed a 54% carbohydrate diet containing 36% starch and 18% sucrose, plus: 10% casein, 10% lactalbumin, 6% cellulose, 8% corn oil, 4% lard, 4% beef tallow, 3.5% salt mix, and 1% vitamin mix. The diet (obtained from Dyets Inc., Bethlehem, PA) provided approximately 49% energy from carbohydrate, 18% from protein, and 33% from fat. Animals were maintained on this diet for 3 months. Body weight was measured monthly in each animal throughout the study. At the end of the feeding period, animals were sacrificed by decapitation. Blood samples were obtained with the animals in the fasting state for 12 h and collected in EDTA and Trasylol for determinations of leptin, glucose, insulin, total cholesterol, and triglycerides in the plasma.

### Analytical Measurements

Plasma glucose concentration was measured by the hexokinase method described by Bondar and Mead.[22] Plasma insulin was measured by radioimmunoassay using the double antibody procedure. Plasma cholesterol and triglyceride concentrations were measured enzymatically in a Centrifichem 600 system (Serono-Baker Diagnostics, Allentown, Pa.). Immunoreactive leptin in plasma was determined by radioimmunoassay using a kit from (Linco Research Inc, St. Charles, MO).

### Statistical Analysis

Results are expressed as mean ± SEM. Comparisons between groups were made using one-way analysis of variance (ANOVA). Differences between mean values in the two groups were tested by Student's *t*-test. Correlations between leptin and the various variables were examined by Spearman correlation analysis (SAS Institute). Differences were considered significant when the p value was $<0.05$.

### RESULTS

All animals progressively gained weight on the 54% carbohydrate diet with the obese group showing considerably higher body weight than the lean group throughout the study. At approximately six weeks on the diet, body weight of obese rats ($n=4$) averaged 427 ± 12 g compared with 362 ± 22 g in lean rats ($n=4$) ($p<0.025$). At this period, mean fasting plasma leptin concentration was 34.1 ± 1.8 μg/l in obese rats and 7.5 ± 1.3 μg/l in lean littermates ($p<0.001$). Fasting plasma insulin levels were also significantly higher in obese than in lean rats with a mean value of 4.46 ± 0.8 nmol/l in the obese group compared with 0.35 ± 0.1 nmol/ml ($p<0.001$).

At the end of 12 weeks on the diet, obese and lean rats showed further increases in body weight, plasma leptin, and plasma insulin (Tab. I). The mean body weight of obese rats was 30% higher than that of their lean littermates ($p<0.0001$). Mean fasting plasma leptin concentration was approximately 8-fold higher in obese than in lean controls ($p<0.01$); whereas, plasma insulin was 6-fold higher in the obese than in the lean group ($p<0.01$). In addition, fasting levels of glucose, cholesterol, and triglycerides were all significantly higher in obese rats than in lean rats.

Spearman correlations between leptin and various parameters in all animals are summarized in Table II. There was wide variability in plasma leptin and plasma insulin levels among the animals. Individual levels of plasma leptin were positively correlated with body weight among all rats ($r=0.73$, $p<0.01$) (Fig. 1). Similarly, plasma insulin concentration was

TABLE I Fasting plasma levels of leptin, insulin, glucose, total cholesterol, and triglyceride in obese and lean SHR/N-*cp* rats

| Parameter | Obese ($n=9$) | Lean ($n=12$) |
|---|---|---|
| Body weight, g | 580 ± 9* | 447 ± 9 |
| Plasma leptin, μg/l | 112 ± 11* | 13.8 ± 3 |
| Plasma insulin, nmol/l | 7.2 ± 2* | 1.2 ± 0.3 |
| Plasma glucose, mmol/l | 9.5 ± 0.5* | 7.5 ± 0.3 |
| Plasma total cholesterol, mmol/l | 4.4 ± 0.4* | 2.9 ± 0.4 |
| Plasma triglycerides, mmol/l | 11.2 ± 3* | 2.4 ± 0.6 |

Values are means ± SE.
*P < 0.01, compared with lean.

TABLE II Spearman correlation coefficients between metabolic variables in SHR/N-*cp* rats

| Variables | Leptin | Insulin | Glucose | Cholesterol | Triglycerides |
|---|---|---|---|---|---|
| Body weight | 0.73** | 0.54* | 0.73* | 0.71* | 0.57* |
| Leptin | – | 0.70** | 0.75* | 0.63* | 0.67* |
| Insulin | – | – | 0.66* | 0.36 | 0.27 |

Number of rats = 21; **p < 0.01; *p < 0.05.

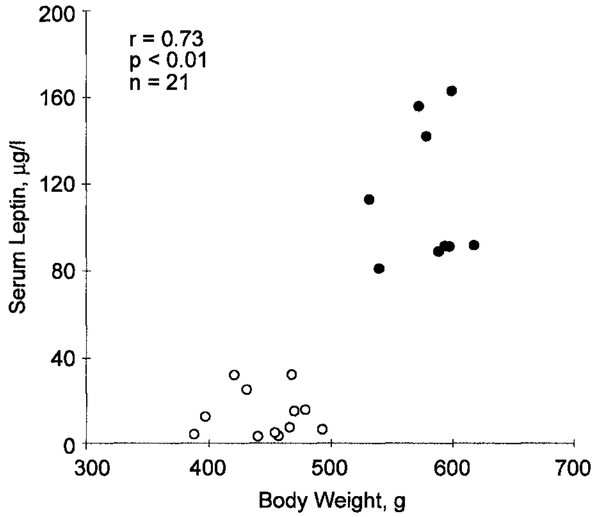

FIGURE 1 Relationship between plasma leptin concentration and body weight in SHR/N-*cp* rats. Open circles represent lean rats (n = 12) and closed circles represent obese rats (n = 9).

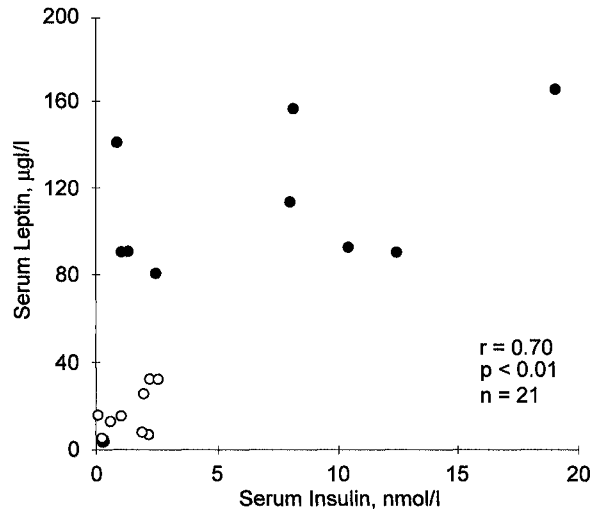

FIGURE 2 Relationship between plasma leptin and plasma insulin in SHR/N-*cp* rats.

significantly correlated with body weight in all animals ($r = 0.54$, $p < 0.05$). There was also a significant positive correlation between plasma leptin and plasma insulin in the entire group ($r = 0.70$, $p < 0.01$) (Fig. 2). However, this direct relationship was significant only for lean rats but not for obese rats ($r = 0.59$, $p < 0.05$ for lean rats, and $r = 0.23$, p = NS, for obese rats). Plasma leptin also correlated significantly with fasting plasma glucose ($r = 0.75$, $p < 0.05$), total cholesterol ($r = 0.63$, $p < 0.05$), and triglyceride ($r = 0.67$, $p < 0.05$).

### DISCUSSION

Our study is the first evaluation of plasma leptin and its relationship to insulin and metabolic parameters in the SHR/N-*cp* rat, a genetic animal model of obesity, NIDDM, and hypertension. Our results demonstrate that plasma leptin levels are consistently higher in obese SHR/N-*cp* rats when compared with their lean littermates. Further, the hyperleptinemia was observed at an early age, *e.g.*, approximately 12 weeks of age, when obese rats already show higher body weight compared with lean rats. Hyperinsulinemia is also observed in obese SHR/N-*cp* rats as early as 4 weeks of age.[23] After 12 weeks of feeding a high carbohydrate diet, both obese and lean rats gained more weight and exhibited 8-fold higher fasting levels of plasma leptin than their lean littermates. However, we noted wide variations in plasma leptin in both lean and obese rats, even though they were all of the same age and gender, and on the same diet. This interindividual variation in leptin may be due to the genetic background of the animals. The lean group comprised of heterozygous (*cp*/+) and homozygous (+/+) lean rats, whereas, the obese group consisted of homozygous (*cp*/*cp*) corpulent rats. This is further suggested by a recent study of Cleary *et al.*[24] in the Zucker fatty rat, which showed that heterozygous (*FA*/*fa*) lean rats had higher levels of plasma leptin when compared with homozygous (*FA*/*FA*) lean rats. In our study, we did not separate heterozygous lean SHR/N-*cp* rats from homozygous lean animals. Nevertheless, individual levels of plasma leptin in the entire group were positively correlated with body weight.

Our results are complementary to previous reports showing a positive correlation between circulating leptin and body fat mass in obese humans as well as in several animal models of obesity.[12, 13, 16] In contrast to findings in the *ob/ob* mouse which lacks the *ob* gene and leptin,[7] the marked elevation of plasma leptin in obese SHR/N-*cp* rats indicates that adiposity in these animals, like that in most other rodent models of obesity, is related to up-regulation of the *ob* gene. Indeed, several studies have shown that ob mRNA levels and leptin secretion are augmented in direct proportion to severity of obesity in diabetic (*db/db*) mice, Wistar fatty rat, and Zucker (*fa/fa*) rats.[14–16] More relevant to the present study is the finding by Hiraoka and co-workers[25] of a marked increase in *ob* gene expression and leptin secretion in the obese SHR or Koletsky rat, the parent strain from which the SHR/N-*cp* rat was originally derived. In their study, plasma leptin levels were found to be 100-fold higher in obese than in lean SHRs, whereas in our study, serum leptin levels were about 8-fold higher in obese SHR/N-*cp* rats compared with their lean littermates. Additionally, Shillabeer *et al.*[26] reported increased mRNA leptin levels in proportion to adipocyte number in JCR:LA-corpulent rats, a substrain which is also derived from obese SHR.

Obese SHR/N-*cp* rats also exhibited marked hyperinsulinemia, mild hyperglycemia, hypercholesterolemia, and hypertriglyceridemia, as has been observed in previous studies.[18, 19] We have previously reported that the hyperinsulinemia in obese rats is associated with marked hyperplasia of pancreatic $\beta$-cells.[20] In the present study, the marked hyperinsulinemia in obese SHR/N-*cp* rats was associated with marked elevations in fasting serum leptin concentrations, suggesting an interaction between these two hormones in the development of obesity. Insulin has recently been suggested to play an important role in the regulation of leptin secretion, in addition to its contributory role in the development of obesity in animals and humans. Studies in rats with insulin deficiency induced by streptozotocin have shown that plasma leptin levels and leptin mRNA are markedly reduced and treatment with insulin in these animals restores leptin levels to normal.[27] Acute and chronic administration of insulin has been shown to increase ob mRNA expression in other experimental animals.[21, 28] Taken together, these observations indicate that plasma leptin concentrations are markedly reduced under conditions of insulin deficiency and are increased by exogenous insulin administration. In our study of the SHR/N-*cp* rat, we also found a direct correlation between plasma leptin and insulin levels. More interestingly, this relationship was more evident in lean than in obese rats, suggesting that the expression of these two hormones is closely linked to their heterozygous *cp* background. On the other hand, homozygous obese SHR/N-*cp* rats with severe hyperinsulinemia have also markedly elevated levels of circulating leptin. Hence, no further increase in plasma leptin level was observed, perhaps, because leptin secretion in the obese animals was already maximally stimulated.

We also observed a significant positive correlation between plasma leptin concentration and fasting levels of plasma glucose, total cholesterol, and triglycerides. These results extend the recent observations by Haffner *et al.*[29] in male non-diabetic human subjects showing a significant correlation of serum leptin levels with whole-body glucose disposal rate (GDR), fasting glucose, total triglycerides, apolipoprotein B, and low density lipoprotein (LDL) size. Similar correlations between serum leptin levels and body fat content, fasting insulin, fasting glucose, and triglycerides have been reported by Iida *et al.*[30] in studies of the Otsuka-Long-Evans Tokushima-Fatty (OLETF) rat, another rodent model of spontaneous NIDDM. Thus, it appears that high circulating leptin may also be associated with metabolic risk factors that are related to insulin resistance.

In contrast to the stimulatory effects of insulin on leptin secretion, leptin has been shown in many studies to inhibit insulin release.[8, 31, 32] This effect of leptin appears to be a direct inhibitory action of the hormone on leptin receptors (ObRb) present in pancreatic $\beta$-cells.[31, 32] This interaction between insulin and leptin has led to the hypothesis recently proposed by Kieffer and Habener[33] that there exists an adipoinsular axis, a dual hormonal feed back loop involving insulin and leptin produced by pancreatic beta cell and adipocytes, respectively. In this schema, insulin, a well-known adipogenic hormone, increases body fat mass and stimulates the production and secretion of leptin that acts centrally to reduce food intake and increase energy expenditure. Leptin in turn suppresses insulin secretion by both central actions and direct actions on pancreatic beta cells. Since circulating levels of leptin are directly proportional to body fat mass, an increase in obesity increases plasma leptin, thereby reducing insulin secretion. However, it should be noted that leptin lowers plasma insulin levels in *ob/ob* mice which genetically lack leptin whereas it has no insulin-lowering effect in *db/db* mice with defective leptin receptors,[31, 32] indicating that the leptin-insulin feedback loop is disrupted in diabesity syndrome in mice with hyperleptinemia. The direct association of plasma insulin with plasma leptin observed in SHR/N-*cp* rats suggests that this hormonal feedback loop is also disrupted in this model. Such failure of this adipoinsular axis to suppress insulin secretion may contribute to the development of adiposity, chronic hyperinsulinemia, and hyperleptinemia in this model of obesity and NIDDM.

In summary, we have shown for the first time that circulating leptin is markedly elevated in obese SHR/N-*cp* rats compared with their lean littermates. Fasting plasma leptin concentration was positively correlated with body weight, suggesting that obesity in this animal model is related to upregulation of the *ob* gene. Plasma insulin was also markedly elevated in obese rats compared with lean rats and was positively correlated with body weight. Furthermore, plasma leptin was significantly correlated with plasma insulin suggesting an interaction between these two hormones in the development of obesity in this model. Variations in steady-state levels of circulating leptin and insulin in lean SHR/N-*cp* rats may reflect heterozygosity of their *cp* gene background. Plasma leptin levels also correlated directly with plasma glucose, plasma cholesterol, and plasma triglycerides. We conclude that circulating leptin may be one of the best biological markers of obesity and that hyperleptinemia is closely associated with several major risk factors known to be related to insulin resistance in the diabesity syndrome.

### *References*

[1] Berchtold, P., Jorgens, V., Finke, C. and Berger, M. (1981). Epidemiology of obesity and hypertension. *Int. J. Obesity*, **5**, 1–7.

[2] Panzram, G. (1987). Mortality and survival in type 2 (non-insulin-dependent) diabetes mellitus. *Diabetologia*, **30**, 123–131.

[3] Zimmet, P. Z. (1992). Kelly West Lecture 1991. Challenges in diabetes epidemiology – from west to the rest. *Diabetes Care*, **15**, 232–252.

[4] Modan, M., Halkin, H., Almog, S., Lusky, A., Eshkol, A., Shefi, M., Shitrit, A. and Fuchs, Z. (1985). Hyperinsulinemia: A link between hypertension, obesity, and glucose intolerance. *J. Clin. Invest.*, **75**, 809–817.

[5] De Fronzo, R. A. and Ferrannini, E. (1991). Insulin resistance: A multifaceted syndrome responsible for NIDDM, obesity, hypertension, dyslipidemia and atherosclerotic cardiovascular disease. *Diabetes Care*, **14**, 173–194.

[6] Olefsky, J. M., Kolterman, O. G. and Scarlett, J. A. (1982). Insulin action and resistance in obesity and non-insulin dependent type II diabetes mellitus. *Am. J. Physiol.*, **243**, E15–E30.

[7] Zhang, Y., Proenca, R., Maffei, M., Barone, M., Leopold, L. and Friedman, J. M. (1994). Positional cloning of the mouse obese gene and its human homologue. *Nature*, **372**, 425–432.

[8] Pelleymounter, M. A., Cullen, M. J., Baker, M. B., Hecht, R., Winters, D., Boone, T. and Collins, F. (1995). Effects of the obese gene product on body weight regulation in ob/ob mice. *Science*, **269**, 540–543.

[9] Halaas, J. L., Gajiwala, K. S., Maffei, M., Cohen, S. L., Chait, B. T., Rabinowitz, D., Lallone, R. L., Burley, S. K. and Friedman, J. M. (1995). Weight-reducing effects of the plasma protein encoded by the obese gene. *Science*, **269**, 543–546.

[10] Weigle, D. S., Bukowski, T. R., Foster, D. C., Kramer, J. M., Lasser, G., Loften-Day, C. E., Prunkard, D. E., Raymond, C. and Kujiper, J. L. (1995). Recombinant ob protein reduces feeding and body weight in the ob/ob mouse. *J. Clin. Invest.*, **96**, 2065–2070.

[11] Considine, R. V., Considine, E. L., Williams, C. J., Nyce, M. R., Magosin, S. A., Bauer, T. L., Rosato, E. L., Colberg, J. and Caro, J. F. (1995). Evidence against either a premature stop codon of the absence of ob gene mRNA in human obesity. *J. Clin. Invest.*, **95**, 2986–2988.

[12] Maffei, M., Halaas, J., Ravussin, E., Pratley, R. E., Lee, G. H., Zhang, Y., Fei, H., Kim, S., Lallone, R., Ranganathan, S., Kern, P. A. and Friedman, J. M. (1995). Leptin levels in human and rodent: measurement of plasma leptin and ob RNA in obese and weight reduced subjects. *Nature Med.*, **11**, 1155–1161.

[13] Considine, R. V., Sinha, M. K., Heiman, M. L., Kriauciunas, A., Stephens, T. W., Nyce, M. R., Ohannesian, J. P., Marco, C. C., McKee, L. J., Bauer, T. L. and Caro, J. F. (1996). Serum immunoreactive-leptin concentrations in normal-weight and obese humans. *New. Engl. J. Med.*, **334**, 292–295.

[14] Chua, S. C., Chung, W. K., Wu-Peng, X. S., Zhang, Y., Liu, S.-M., Tartaglia, L. and Liebel, R. L. (1996). Phenotypes of mouse diabetes and rat fatty due to mutations in the OB (leptin) receptor. *Science*, **27**, 994–996.

[15] Ogawa, Y., Masuzaki, H., Isse, N., Okasaki, T., Mori, K., Shigemoto, M., Satoh, N., Tamura, N., Hosoda, K., Yoshimasa, Y., Jingami, H., Kawada, T. and Nakao, K. (1995). Molecular cloning of rat obese cDNA and augmented gene expression in genetically obese Zucker fatty (fa/fa) rats. *J. Clin. Invest.*, **96**, 1647–1652.

[16] Frederich, R. B., Lollman, B., Hamman, A., Napolitano-Rosen, A., Kahn, B. B., Lowell, B. B. and Flier, J. S. (1995). Expression of Ob mRNA and its encoded protein in rodents. *J. Clin. Invest.*, **96**, 1658–1663.

[17] Maffei, M., Fei, H., Lee, G. H., Dani, C., Leroy, P., Zhang, Y., Proenca, R., Negrel, R., Ailhaud, G. and Friedman, J. M. (1995). Increased expression in adipocytes of ob mRNA in mice with lesions of the hypothalamus and with mutations at the db locus. *Proc. Natl. Acad. Sci. USA*, **92**, 6957–6960.

[18] Michaelis IV, O. E., Ellwood, K. C., Judge, J. M., Schoene, N. W. and Hansen, C. T. (1984). Effect of dietary sucrose on the SHR/N-corpulent rat: a new model for insulin-independent diabetes. *Am. J. Clin. Nutr.*, **39**, 612–618.

[19] Michaelis IV, O. E., Patrick, D. H., Hansen, C. T., Canary, J. J., Werner, R. M. and Carswell, N. (1986). Spontaneous hypertensive/NIH-corpulent rat. Animal model for insulin-independent diabetes mellitus (Type II). *Am. J. Path*, **123**, 398–400.

[20] Michaelis IV, O. E., Carswell, N., Hansen, C. T., Canary, J. J. and Kimmel, P. L. (1988). A new genetic model of noninsulin-dependent diabetes and hypertension: the spontaneous hypertensive/NIH corpulent rat. In *Frontiers in Diabetes Research*, Edited by Shafrir, E. and Renold, E. A., pp. 257–264. John Libbey & Company, Ltd.

[21] Cusin, I., Sainsbury, A., Doyle, P. and Rohner-Jeanrenaud, B. (1995). The ob gene and insulin: a relationship leading to the understanding of obesity. *Diabetes*, **44**, 1467–1470.

[22] Bondar, R. J. and Mead, D. C. (1974). Evaluation of glucose-6-phosphate dehydrogenase from leuconostoc mesenteroides in the hexokinase method for determining glucose in the serum. *Clin. Chem.*, **20**, 586–590.

[23] Voyles, N. R., Powell, A. M., Timmers, K. I., Wilkins, S. D., Bhathena, S. J., Hansen, C., Michaelis IV, O. E. and Recant, L. (1988). Reversible impairment of glucose-induced insulin secretion in SHR/N-cp rats. Genetic model of type II diabetes. *Diabetes*, **37**, 398–404.

[24] Cleary, M. P. and Phillips, F. C. (1999). The presence of the "fa" gene in the heterozygous (FA/fa) lean female rats: effects on body weight, body fat and serum leptin. *Obes. Res.*, **7**, 293–298.

[25] Hiraoka, J., Hosoda, K., Ogawa, Y., Ikeda, K., Nara, Y., Masuzaki, H., Takaya, K., Nagakawa, K., Mashimo, T., Sawamura, M., Koletsky, R. J., Yamori, Y. and Nakao, K. (1997). Augmentation of obese (ob) gene and leptin secretion in obese spontaneously hypertensive rats (obese SHR or Koletsky rats). *Biochem. Biophys. Res. Commun.*, **231**, 582–585.

[26] Shillabeer, G., Vydelingum, S., Hatch, G., Russell, J. C. and Lau, D. C. (1998). Long-term regulation of leptin secretion is correlated with adipocyte number in obese rats. *Clin. Invest. MED.*, **21**, 54–62.

[27] Sivitz, W. I., Walsh, S., Morgan, D., Donohoue, P., Haynes, W. and Leibel, R. L. (1998). Plasma leptin in diabetic and insulin-treated diabetic and normal rats. *Metabolism*, **47**, 584–591.

[28] Saladin, R., De Vos, P., Guerre-Millo, M., Leturque, A., Girard, J., Staels, B. and Auwerx, J. (1995). Transient increase in obese gene expression after food intake and insulin administration. *Nature*, **377**, 527–529.

[29] Haffner, S. M. Mykkanen, L., Rainwater, D. L., Karhapaa, P. and Laakso, M. (1999). Is leptin concentration associated with the insulin resistance syndrome in nondiabetic men. *Obese. Res.*, **7**, 164–169.

[30] Iida, M., Murakami, T., Sei, M., Kuwajima, M., Yamada, M., Aono, T. and Shima, K. (1998). Circulating leptin did not associate with the development of the hyperglycemia accompanied by insulin sensitivity in spontaneous noninsulin dependent diabetes mellitus model Otsuka-Long-Evans-Tokushima-Fatty rats. *Regul. Pept.*, **77**, 141–146.

[31] Kulkarni, R. N., Wang, G. L., Wang, R. M., Hurley, J. D., Smith, D. M., Ghatei, M. A., Whithers, D. J., Gardiner, J. V., Bailey, C. J. and Bloom, S. R. (1997). Leptin rapidly suppresses insulin release from insulinoma cells, rat and human islets and, *in vivo*, in mice. *J. Clin. Invest.*, **100**, 2729–2736.

[32] Emilsson, V., Liu, Y. L., Cawthorne, M. A., Morton, N. M. and Davenport, M. (1997). Expression of the functional leptin receptor mRNA in pancreatic islets and direct inhibitory action of leptin on insulin secretion. *Diabetes*, **46**, 313–316.

[33] Kieffer, T. J. and Habener, J. F. (2000). The adipoinsular axis: effects of leptin on pancreatic $\beta$-cells. *Am. J. Physiol. Endocrinol. Metab.*, **278**, E1–E14.

